# Fungal Kti12 proteins display unusual linker regions and unique ATPase p-loops

**DOI:** 10.1007/s00294-020-01070-2

**Published:** 2020-03-31

**Authors:** Rościsław Krutyhołowa, Annekathrin Reinhardt-Tews, Andrzej Chramiec-Głąbik, Karin D. Breunig, Sebastian Glatt

**Affiliations:** 1 Malopolska Centre of Biotechnology (MCB), Jagiellonian University, Krakow, Poland; 2 Faculty of Biochemistry, Biophysics and Biotechnology, Jagiellonian University, Krakow, Poland; 3 Institut für Biologie, Martin-Luther-Universität Halle-Wittenberg, Halle (Saale), Germany

**Keywords:** Kti12, PSTK, Flexible linker, Active site, P-loop, ATPase

## Abstract

Kti12 (*Kluyveromyces lactis* toxin insensitive 12) is an evolutionary highly conserved ATPase, crucial for the tRNA-modification activity of the eukaryotic Elongator complex. The protein consists of an N-terminal ATPase and a C-terminal tRNA-binding domain, which are connected by a flexible linker. The precise role of the linker region and its involvement in the communication between the two domains and their activities remain elusive. Here, we analyzed all available Kti12 protein sequences and report the discovery of a subset of Kti12 proteins with abnormally long linker regions. These Kti12 proteins are characterized by a co-occurring lysine to leucine substitution in their Walker A motif, previously thought to be invariable. We show that the K14L substitution lowers the affinity to ATP, but does not affect the catalytic activity of Kti12 at high ATP concentrations. We compare the activity of mutated variants of Kti12 in vitro with complementation assays in vivo in yeast. Ultimately, we compared Kti12 to other known p-loop ATPase family members known to carry a similar deviant Walker A motif. Our data establish Kti12 of *Eurotiomycetes* as an example of eukaryotic ATPase harboring a significantly deviating but still functional Walker A motif.

**Electronic supplementary material:**

The online version of this article (10.1007/s00294-020-01070-2) contains supplementary material, which is available to authorized users.

## Introduction

It was noticed several years ago that yeast cells become resistant to *Kluyveromyces lactis* toxin, zymocin, upon removal of any of the so-called KTI genes (Butler et al. 1991b; 1994; Kishida et al. 1996). The family of Kti protein harbors various types of proteins, including Elongator core subunits (Elp1/Kti7, Elp2/Kti3, Elp3/Kti8, Elp4/Kti9, Elp6/Kti4), methyltransferases (Trm9/Kti1), zinc-binding proteins (Kti11), WD40s (Kti13), and kinases (Hrr25/Kti14) (Frohloff et al. 2001; Huang et al. 2005a; Karlsborn et al. 2014; Krutyhołowa et al. 2019b). Zymocin itself has three individual components, namely alpha-, beta- and gamma subunits (Stark and Boyd 1986). Alpha- and beta-subunits serve as auxiliary factors allowing the delivery of the gamma subunit into fungal cells (Butler et al. 1991a). The gamma subunit cleaves cellular tRNAs that specifically carry a double 5-methoxy-carbonyl-methyl-2-thio (mcm^5^s^2^U_34_) modification at uridines in their wobble position (Lu et al. 2005; Jablonowski et al. 2006).

Those chemically rather complicated tRNA modifications are introduced by a cascade of enzymes, including the Elongator complex (Huang et al. 2005b; Dauden et al. 2018), methyltransferases Trm9-Trm112/ALKBH8 (Studte et al. 2008; Songe-Møller et al. 2010; van Tran et al. 2018), and the Uba4/Urm1 thiolation pathway (Leidel et al. 2009; Noma et al. 2009). Despite the creation of a toxin-target site, these tRNA modifications are genuinely required for maintaining proper translation speed and preventing from co-translational protein misfolding (Nedialkova and Leidel 2015; Ranjan and Rodnina 2016; Goffena et al. 2018). The lack of these anticodon modifications in humans causes various pathophysiologies, including severe neurodegenerative diseases (Close et al. 2006; Chaverra et al. 2017; Dauden et al. 2018; Kojic et al. 2018; Hawer et al. 2018; Shaheen et al. 2019).

**Fig. 1:**
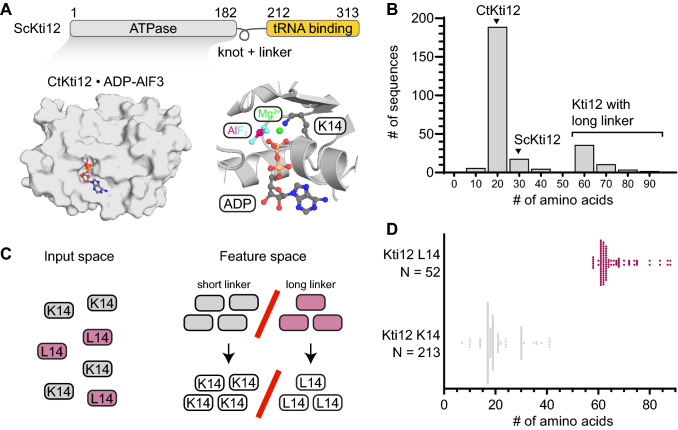
Identification of Kti12 proteins with elongated linker and their characterization. **a** Architecture of Kti12 protein. ATPase domain (grey) and tRNA-tethering domain (yellow) are connected by a flexible linker. Structural overview of *Chaetomium thermophilum* Kti12 ATPase domain (PDB ID: 6QP0) with a particular emphasis on nucleotide binding pocket and K14 located in a p-loop. **b** Distribution of linker length across fungal Kti12 proteins. Numbers on *O*_x_ indicate middle values for particular baskets, for instance, 18 ± 4. Black triangles indicate groups to which *Sc*Kti12 and *Ct*Kti12 belong to. **c** Scheme of supervised a machine-learning experiment that pinpointed coincidence of L14 with elongated linkers. Sequences with linkers of a regular length (grey) always carry lysine at position 14 (K14), whereas Kti12 proteins with elongated linkers (pink) can be distinguished by the presence of leucine in a p-loop (L14). **d** Violin plots represents distribution of Kti12 protein sequences in a two-dimensional feature space. Linker length is on *O*_x_ whereas presence of leucine or lysine qualifies sample to one of the groups on *O*_y_ axis. Number of sequences within each group is indicated next the *O*_y_ axis

Recent structural analyses of the enzymatic Elp3 subunit and the Elp123 subcomplex of Elongator show that the subcomplex specifically recognizes tRNAs (Glatt et al. 2016; Dauden et al. 2019; Lin et al. 2019). The overall complex consists of six pairs of subunits and is built up by a symmetrical dimer of two Elp123 subcomplexes, decorated with a hexameric Elp456 ring (Glatt et al. 2012) on one of the Elp123 lobes (Dauden et al. 2017; Setiaputra et al. 2017). Kti12 was demonstrated to bind to the Elongator complex in vivo and in vitro (Fichtner et al. 2002; Petrakis et al. 2005), and we recently fine-mapped its binding site to the first WD40 domain of the Elp1 subunit in vitro (Krutyhołowa et al. 2019a). Kti12 harbors two domains, namely an N-terminal ATPase domain and a C-terminal tRNA-binding domain, both connected by a flexible linker region (Fig. 1a) (Schaffrath et al. 2017; Krutyhołowa et al. 2019a). Kti12 shows a very high overall sequence and structural similarity to O-phosphoseryl tRNA^Sec^ kinase (PSTK), a kinase responsible for phosphorylation of Ser-tRNA^Sec^ (Sherrer et al. 2008). Like PSTK (Chiba et al. 2010), Kti12 binds tRNA with its C-terminus and hydrolyzes ATP, if presented with tRNA^Sec^ in vitro (Krutyhołowa et al. 2019a). Other atypical kinases, like the p-loop-containing Rio1 ATPases, are known to play key roles in ribosome biogenesis, translational control, and other cellular functions (Berto et al. 2019). However, the genuine function of Kti12 and the mechanistic consequences of its direct association with the Elongator complex remain elusive (Krutyhołowa et al. 2019a).

Here, we systematically analyzed all annotated Kti12 and PSTK sequences and identified a subpopulation of Kti12 proteins in *Eurotiomycetes* with extremely elongated linker regions. Furthermore, we used a machine-learning approach to unbiasedly identify a co-occurring feature specific to those Kti12 proteins with elongated linkers. In detail, those aberrant Kti12 proteins share a substitution of a highly conserved p-loop lysine, which was previously described as an invariable element of the phosphate-binding motif in ATPases. We investigated this evolutionary coupling and dissected its potential implications for the active site of Kti12. Our study presents complementary approaches in vitro and in vivo that deepen our understanding of Kti12 and provide important insights into the variations of p-loop-based active sites.

## Materials and methods

### Sequence analyses and machine learning

Annotated and validated sequences of Kti12 proteins from different domains of life were obtained from the Uniprot database and aligned using Muscle web server (Edgar 2004) and Jalview software (Waterhouse et al. 2009). Alignments were manually curated, and all proteins without both ATPase domain and CTD were removed. We utilized Python, Biopython library (Cock et al. 2009), and elements of scikit-learn platform (Pedregosa et al. 2011) to conduct a machine-learning experiment. The length of the linker between ATPase domain (NTD) and tRNA-binding domain (CTD) was used for supervised learning. After randomization of multiple sequence alignment, single amino acid positions were considered as a basis for supporting vector machine classification. The significance of a linker-length difference between K14- and L14-containing groups was additionally tested using unpaired two-tailed *t* test and resulted in a *p* value of < 0.0001.

### Modeling and structure visualization

The Kti12 protein structure was visualized using PyMol software (Schrödinger 2015). We used the YRB script (Hagemans et al. 2015) to analyze hydrophobicity of the nucleotide-binding pocket. Homology-based models of mu gp28 were obtained using Phyre 2.0 and *An*Kti12 was modelled using SWISS-MODEL web server (Kelley and Sternberg 2009; Waterhouse et al. 2018). We employed the I-TASSER server (Roy et al. 2010; Yang et al. 2015) to predict the existence of any stable secondary structure elements within the linker of *An*Kti12.

### Strains and genetic manipulation of *Saccharomyces cerevisiae*

The influence of mutations on Kti12 activity was analyzed by gene replacement technology, which allows to modify specific yeast genomic loci (Rothstein 1991). The *S. cerevisiae* strains used and generated are listed (see Table S1). First, the genomic *KTI12* gene of strain UMY2893 (Huang et al. 2005a) was disrupted by transformation with a DNA fragment containing the *URA3* gene from *K. lactis* flanked by *KTI12* homologous regions. Selection for Ura + colonies and screening for zymocin resistance yielded *kti12::KlURA3* transformants, in which the WT gene was replaced with the disrupted allele by a double crossover event in vivo. The resulting strain CKY40 was used in the second step to introduce each of the mutations by generating the corresponding DNA fragments with the desired mutation restoring the *KTI12* locus except for the desired changes. The corresponding DNA fragments were generated by PCR and transformed directly. Replacement of the *KlURA3* cassette by the incoming DNA yielded ura3-auxotrophs selected by plating on 5-fluoroorotic acid-containing media. The proper recombination event was verified by screening colonies using PCR. The presence of the respective mutation was confirmed by sequencing of genomic DNA.

### In vivo assay for Kti12 functionality by yeast phenotyping

To analyze the influence of the different *kti12-*alleles on tRNA modification by the Elongator, growth inhibition by exogenous zymocin toxin was assayed as previously described (Frohloff et al. 2001). Zymocin was obtained from the culture medium of *Kluyveromyces lactis* killer strain AWJ137 (Frohloff et al. 2001) by filtration (Klassen et al. 2011). Tenfold serial dilutions of the different strains were spotted on rich media plates (YPD, 0.5% yeast extract, 2% peptone, 2% glucose and 2% agar) containing 50% (v/v) zymocin or without the toxin and incubated for 2–3 days at 30°C. Functionally intact Kti12 confers growth inhibition due to the cleavage of three Elongator-modified tRNAs by zymocin. Functionally impaired Kti12 causes toxin resistance (Frohloff et al. 2001). In the second assay, the Kti12 function was tested via the nonsense suppression activity of the *SUP4* tRNA, which requires the U_34_ modification in the anticodon. The *kti12* mutations were transferred to the *SUP4* background and the resulting strains, which contain the *ade2-1* or the *can1-100* allele containing ochre stop codons in the coding regions, were spotted on SD plates containing adenine (YNB), lacking adenine (Ade-), or medium without arginine but containing the toxic arginine analog canavanine. Plates were incubated for 3 days at room temperature and 3 days at 30°C. In cells lacking Kti12 activity, nonsense suppression of the *ade2-1* mutation and, therefore, growth without adenine supplementation is impaired. No growth on adenine plates correlates with the growth on canavanine plates since the lack of nonsense suppression of the *can1-100* allele means that the *CAN1*-encoded arginine permease, responsible for the uptake of canavanine, is not expressed. Hence a functional *KTI12* gene product is indicated by growth on Ade^−^ and no growth on canavanine plates. (Huang et al. 2005b).

### Protein purification

The codon optimized open reading frames (ORF), encoding *Chaetomium thermophilum* Kti12 (*Ct*Kti12) or *Aspergillus niger* (*An*Kti12), were individually cloned into the pET24d vector using NcoI and XhoI restriction sites and purified as previously described (Krutyhołowa et al. 2019a). *An*Kti12 protein was additionally purified in a HEPES pH 7.2 buffer using a cation-exchange Hi-Trap SP column (GE Healthcare), followed by size-exclusion chromatography on HiLoad 16/600 Superdex 75 pg column (GE Healthcare). Proteins were stored at – 80 °C in 20 mM Tris–HCl pH 7.5, 150 mM NaCl, 2 mM DTT buffer.

### Thermal shift assay

Thermostability of *Ct*Kti12 WT protein and *Ct*Kti12 mutants was assessed using thermal shift assays. In short, storage buffer with 2 mM MgCl_2_ without nucleotides or with addition of 1 mM ADP or ATP was used to dissect the influence of nucleotide binding on protein stability. 10 µg of protein was heated up from 4 to 98°C at a speed of 0.6°C/min in the presence of SYPRO Orange (Sigma Aldrich) hydrophobic fluorescent dye. Data represent three independent experiments conducted in at least 2 technical replicas.

### Determination of nucleotide-binding affinity by fluorescence polarization

Dissociation constant of MANT-labeled ATP was determined using fluorescence polarization. Experiments were performed in a storage buffer after addition of 2 mM MgCl_2_. Serial twofold dilutions of *Ct*Kti12 ranging from 100 μM (highest concentration) were prepared and mixed with 1 μM MANT-ATP (final concentration). Fluorescence was elicited using 340-nm excitation light and measured at 448 nm. Fluorescence polarization was measured using FlexStation 3 Multi-Mode Microplate Reader (Molecular Devices) with a gain factor of 1000 and at a system temperature of 37°C for the whole duration of the experiment. Data originate from three independent experiments. Curve fitting and *K*_d_ calculation were conducted using GraphPad Prism software (La Jolla, California, USA; www.graphpad.com) assuming a single/one-site binding mode.

### Production of tRNA

Human tRNA^Sec^ was obtained by run-off in vitro transcription from a linearized pUC 19 vector carrying tRNA^Sec^ gene under the T7 promoter. After overnight transcription reaction, RNA species were separated on a HiTrap DEAE FF column (GE Healthcare). RNA was precipitated with 1:1 v/v isopropanol overnight and subsequently denatured and refolded by heating to 80 °C and cooling to 40 °C in annealing buffer, which contained 200 mM HEPES pH 7.5, 500 mM KCl, and 500 mM NaCl. Subsequent size-exclusion chromatography on Superdex 75 10/300 GL column yielded a homogeneous population of folded tRNA^Sec^ molecules that were used to elicit ATPase activity of Kti12.

### ATPase activity measurements

In vitro transcribed tRNA was used to trigger the ATPase activity of different *Ct*Kti12 protein variants. Reactions were performed in the protein storage buffer after the addition of 2 mM MgCl_2_. In detail, 1 μM *Ct*Kti12 was mixed with 1 μM human tRNA^Sec^ and either 1 mM ATP or 20 μM ATP. Reactions were incubated for 15 h or 1 h, respectively. In the case of *An*Kti12, the reaction was conducted in the presence of 5 μM tRNA^Sec^ for 2 h at 37 °C. Reactions were monitored using malachite green ATPase assay kit (Sigma Aldrich) as per the manufacturer’s instructions. Data derived from at least three independent experiments were performed in technical duplicates.

## Results

### Active site mutation evolutionarily coincides with elongated linker

We harvested and aligned all annotated Kti12 protein sequences from animals, plants, and fungi to identify conserved and variable features in its domains and motifs. Protein sequences of O-phosphoseryl tRNA^Sec^ kinase (PSTK) from animals, which show almost identical domain organization and structural similarity to Kti12, served as references in all analyses (Fig. S1A). First, we systematically compared the length of the flexible linker region that connects the ATPase domains with the C-terminal tRNA-binding domains of Kti12 and PSTK. In animals, Kti12 and PSTK both exhibit relatively short linker regions in the range 18–22 amino acids (Fig. S1B), whereas Kti12 proteins in plants possess longer linkers of approximately 30 amino acids (Fig. S1B). Fungal Kti12 proteins have predominantly shorter linkers of an “animal type”, but some fungi, including *Saccharomyces cerevisiae*, harbor long plant-like linkers. Unexpectedly, some fungal species evolved a third, clearly separated, population of Kti12 proteins with extremely elongated linkers of approximately 55–60 amino acids (Fig. 1b).

**Fig. 2:**
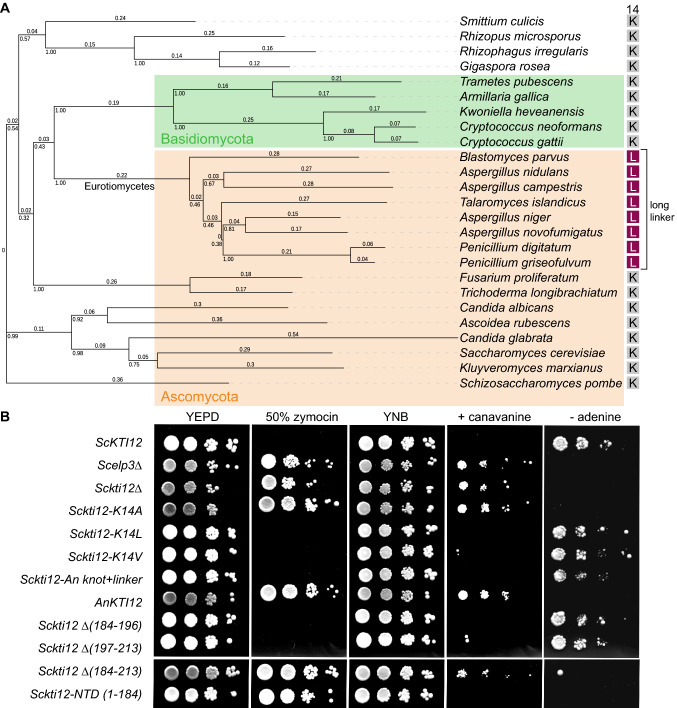
Phenotypic characterization of Kti12 K14L mutation and elongated linker in yeast. **a** Phylogenetic tree based on Kti12 sequence alignment displays distribution of different residues at position 14 among species of different divisions [Ascomycota (orange)/Basidiomycota (green)]. Kti12 proteins carrying L14 and long linker (purple squares) belong to *Eurothiomycetes*. Multiple sequence alignment of Kti12 proteins from 25 model fungi species was performed using JalView software (Waterhouse et al. 2009) and Muscle server (Edgar 2004). The phylogenetic tree was built using the neighbor-Joining method. Individual branch lengths are shown above the branches and indicate the number of amino acid substitutions per site. The fraction of replicate trees in which the associated taxa clustered together in the bootstrap test [1000 replicates (Felsenstein 1985)] are shown beneath the branching points. The evolutionary distances used to calculate the tree were calculated using the Poisson correction method with the pairwise deletion of ambiguous positions. Final dataset contained 610 positions. All evolutionary analyses were conducted in MEGA X (Kumar et al. 2018). The tree was visualized using iTOL (Letunic and Bork 2007). **b** Zymocin assay assessing performance of the Elongator complex and presence of U_34_ modifications. WT yeast strain (ScKTI12) served as a growth control, *Scelp3Δ* and *Sckti12Δ* pose negative controls. Tenfold dilutions were spotted on YEPD media with the addition of 50% Zymocin or YNB media replacing arginine by 40 µg/ml canavanine or lacking adenine (-adenine). In the different strains *KTI12* was mutated (K14A/L), parts or the whole gene were exchanged with the corresponding sequences from *Aspergillus niger* (*An* knot + linker, *An*KTI12) or parts of it were deleted (marked with ∆ and the deleted residues in brackets). *Sckti12-NTD* codes for the first 184 amino acids

We utilized a machine-learning approach to dissect potential commonalities in the case of fungal Kti12 proteins with an elongated linker and understand what distinguishes this population from fungal Kti12 proteins with linkers of typical lengths. First, we manually curated alignments and separated sequences with elongated linkers from other fungal Kti12 sequences. This dataset was used to run a supervised machine-learning approach based on Support Vector Machine (SVM) (Fig. 1c), where we considered single amino acid positions in a multiple sequence alignment and tested if they led to a similar classification pattern. Surprisingly, SVM could achieve a completely equal classification pattern (*r*=1, accuracy=100%) based on the identity of a single amino acid, namely a central active site lysine residue (K14, Fig. 1d). Other identified single amino acid hallmarks of long linker Kti12 proteins neither separate the classes fully nor are located within the Kti12 active site (Fig. S1C). The identified lysine residue was previously demonstrated to be of major importance for the ATPase activity of Kti12, as a K14A mutant showed almost completely abolished ATPase activity in vitro and phenocopied Kti12- and Elongator-deficient *S. cerevisiae* strains (Krutyhołowa et al. 2019a). Strikingly, all sequences with an elongated linker carry leucine or isoleucine residues at the respective position. An additional analysis of the amino acid content of the linker regions revealed that long linkers, in general, harbor more threonine residues, while having noticeably decreased relative number of lysine residues (Fig. S1D). It remains to be shown, if specific residues in the linker could influence the active site of Kti12 proteins carrying the K14L substitution. Species carrying Kti12_L/I14_ belong to the class of *Eurotiomycetes* in *Ascomycota* (Fig. 2a). Inactivating Kti12 mutations would most likely also compromise Elongator’s tRNA modification activity (Schaffrath et al. 2017) and reduce the selective pressure to preserve a functional Elongator complex. Nonetheless, no reports indicate malfunctioning of Elongator or other tRNA modification pathways in *Eurotiomycetes*. Furthermore, we found that all six Elongator genes (Elp1–6) are present in several analyzed *Eurotiomycetes* species and do not contain any genomic alterations (e.g. internal stop codons, fused gene products or frameshift mutations) that would indicate a loss-of-function. The question immediately arises whether *Eurotiomycetes* that carry both Kti12_L/I14_ and an elongated linker have lost the regulatory link between Elongator and the ATPase activity of Kti12 or if the naturally occurring Kti12 variants remain active.

### Both K14L and long linker ScKti12 rescue the *kti*12Δ phenotype

As described previously, K14 of *Sc*Kti12 is of profound importance to proper functioning of Kti12 protein (Krutyhołowa et al. 2019a). Thus, we decided to experimentally verify if these variations in *KTI*12 alleles would have any functional relevance and if the linker length is indeed functionally coupled to alterations in the ATP-binding pocket. Therefore, Kti12 function was tested in *S. cerevisiae* by established assays for functional Elongator-dependent tRNA modifications. The degree of U_34_ modifications in vivo can roughly be estimated by zymocin sensitivity and suppression of ochre stop codons by the SUP4 suppressor tRNA (Frohloff et al. 2001; Huang et al. 2005a). The phenotypes of the generated *Sckti12*–*K14L* mutant resemble the WT (*ScKTI12*) contrasting with the *Sckti12*–*K14*A mutants (Fig. 2b), which phenocopy the null mutant lacking Kti12 protein *(Sckti12Δ)* or lacking the Elongator subunit *Scelp3Δ* (Krutyhołowa et al. 2019a). Sensitivity to zymocin and canavanine are semi-quantitative indicators of Kti12 function. Growth on media lacking adenine is complementary but sometimes less reliable due to the appearance of revertants. The phenotypes indicate that not only the K14L mutation but also shorter deletions of the N- or C-terminal part of the linker (Δ184-196 and Δ197-213) did not substantially affect Kti12 function negatively. Replacing the short linker of *Sc*Kti12 with an elongated linker from *Aspergillus niger* Kti12 (*Sckti12*–*AnKnoLi*) resulted in a variable phenotype on media deprived of adenine suggesting weaker Kti12 activity. Complete deletion of the linker (Δ184-213) inactivates ScKti12. The entire *AnKTI12* gene from *Aspergillus niger* was unable to replace the function of the ScKt12, which might occur due to specific-interaction interfaces with the Elongator complex or other binding partners. Our data illustrate that the linker region in Kti12 is as important for the tRNA-modification activity of Elongator as the N-terminal ATPase domain or the C-terminal tRNA-binding domain (Schaffrath et al. 2017).

### Mutation of K14 alters nucleotide binding by Kti12

**Fig. 3:**
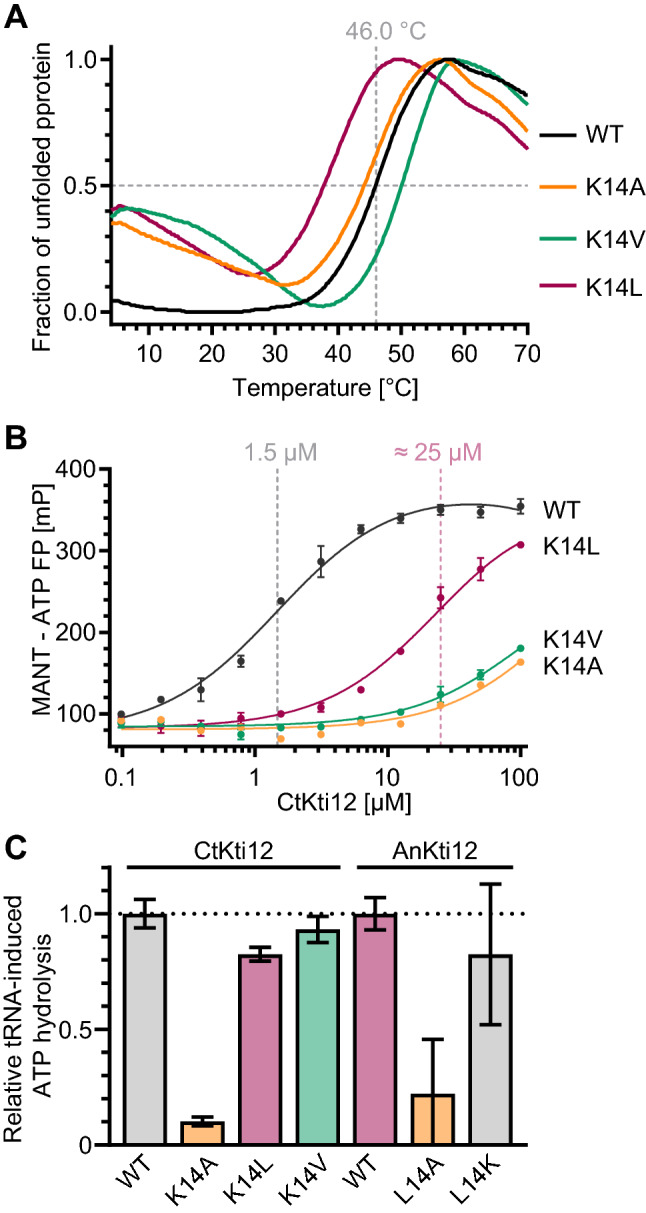
Biochemical properties of Kti12 mutants. **a** Thermostability profiles of CtKti12 K14 point mutants. Thermal shift assay, curves represent an average of three independent experiments. **b** Fluorescent polarization study of MANT-ATP bound by Kti12 mutants. CtKti12 WT Kd = 1.5 μM ± 0.2 μM, K14L Kd = 25.0 μM ± 2.4 μM. In case of CtKti12 K14A and K14V we were unable to determine *K*_d_ in a measured concentration range. **c** Relative tRNA^Sec^-induced ATPase activity of *Ct*Kti12 and *An*Kti12 mutants, compared to the respective WT versions. Free phosphate concentration was measured using malachite green assay. Bars represent an average of three independent experiments ± standard deviation. Proteins carrying different amino acids at position 14 are color coded (K14 grey, A14 orange, L14 purple and V14 green)

Due to the poor solubility of active site mutants from *S. cerevisiae* Kti12 (*Sc*Kti12), we expressed and purified full-length *Chaetomium thermophilum Ct*Kti12 and the variants *Ct*K12_K14A_ and *Ct*Kti12_K14L_ in bacteria (Fig. S2A, B). In addition, we generated another variant, namely *Ct*Kti12_K14V_, which contains a side chain with an intermediate length, allowing us to define the minimal spatial requirements for protein activity. Next, we studied thermostability, nucleotide binding, and tRNA-induced enzymatic ATP hydrolysis activity of *Ct*Kti12 and its variants at saturating and semi-saturating substrate concentrations. *Ct*Kti12 exhibits a melting point of ~ 46.0 °C (Fig. 3a), which increases to 50.0 °C upon addition of ATP. As previously shown (Krutyhołowa et al. 2019a), the substitution K14A does not affect the thermostability of *Ct*Kti12 by itself, but diminishes any nucleotide-dependent stabilization effects. The Kti12_K14V_ mutated variant displays a thermostability profile similar to the nucleotide-bound *Ct*Kti12, even in the absence of a nucleotide, suggesting a dynamic rearrangement of the K14 carbon side chain during nucleotide binding. In contrast, the Kti12_K14L_ variant is strongly destabilized, resulting in a decreased melting temperature of 37.5 °C, 8.5 °C less than the WT. Interestingly, neither K14V nor K14L exhibited ATP-dependent increase of the melting temperature, indicating strongly decreased nucleotide-binding properties of these p-loop variants (Fig. S2C), similar to K14A.

Although the thermal shift assay provided some initial indications regarding nucleotide binding, we decided to quantify nucleotide-binding affinity using fluorescence polarization. Using this method, we were able to confirm the previously measured *K*_d_ for *Ct*Kti12 WT and MANT-ATP (1.5 ± 0.2 μM). Apparently a very high evolutionary constraint conserved the lysine residue in the p-loop motif of yeast Kti12, PSTK, and other family members. Both K14A and K14V proteins have very low affinity towards MANT-ATP and the *K*_d_ can only be estimated to values higher than 100 μM. Interestingly, the naturally occurring K14L variant can still bind MANT-ATP (*K*_d_ 25.0 ± 2.4 μM). In summary, our results demonstrate that all tested substitutions of K14 decrease the affinity towards ATP (Fig. 3b).

Next, we examined the activity of KTI12 variants using the previously established ATPase activity assay (Krutyhołowa et al. 2019a). We observed comparable ATP hydrolysis rates in the case of *Ct*Kti12 WT, K14V, and K14L presented with physiological concentrations of ATP (1 mM; Fig. 3c). This finding supports our in vivo data showing that the K14L variant is able to rescue the *S. cerevisiae Δkti12* strain. In addition, we decided to perform the same assay at more limiting ATP concentration close to the *K*_d_ of the *Ct*Kti12_K14L_-MANT-ATP interaction. Upon incubation with 20 μM ATP, differences in ATP hydrolysis rates between the naturally occurring mutants become unambiguous (Fig. S2D). To further validate our findings from *Ct*Kti12, we expressed and purified WT *Aspergillus niger* Kti12 protein and its L14K and L14A mutants (Fig. S2E). The tRNA-induced ATPase activity of WT *An*Kti12 naturally carrying leucine at position 14 is similar to the ATPase activity of AnKti12_L14K_, the common variant occurring in all other Kti12 proteins. In addition, *An*Kti12_L14A_ shows strongly reduced ATPase activity which further corroborates the observed decrease in ATPase activity of *Ct*Kti12_K14A_ and in vivo phenotyping results of the* Sckti12*–*K14*A strain. Our findings indicate that residues in the long threonine-rich linker of *An*Kti12 cannot contribute to the active site, since the activity of *An*Kti12_L14A_ remains strongly reduced in vitro.

## Discussion

Three different types of linkers are present in Kti12 proteins from fungi and the reason for this divergence currently remains unclear. In the case of animal Kti12 and PSTK, a relatively short linker can be explained by the presence of tRNA^Sec^ which stimulates ATP hydrolysis by Kti12. However, apart from tRNA^Sec^, which does not exist in *S. cerevisiae*, *Sc*Kti12 is important for processing of 11 different tRNAs by the Elongator complex, so the relatively short 20 amino acid linkers may be preferred for tRNA-related functions. Concomitant with this, plant phenotypes caused by mutations in the *Arabidopsis thaliana* deformed root and leaves (DRL1) gene encoding the Kti12 homologue are similar to the phenotypes of elongata (ELO) mutants (Chen et al. 2006; Mehlgarten et al. 2010). Most of the plant genes encoding Elongator core subunits were also identified as *elo* mutants (Nelissen et al. 2005). All plants and some fungi, including *S. cerevisiae*, harbor an approximately 30-amino acid long linker composed of a putative topological knot and a disordered linker. We tried to dissect the role of these regions in *S. cerevisiae* by deleting individual structural elements and respective combinations in the *ScKTI12* gene, but we failed to find any phenotypical difference in the wild type strains lacking the knot or the disordered linker (Fig. 2b). Complete removal of both elements, i.e. the entire linker, inactivated Kti12, whereas replacement of the ScKti12 linker by the long one of AnKti12 did not. Hence, we conclude that cooperation of the ATPase and tRNA-binding domains as described previously (Krutyhołowa et al. 2019a) is supported by a linker, which has properties found in the long *An*Kti12 as well as in the shorter *Sc*Kti12 linker. Attempts to model the structure of the *An*Kti12 linker by I-TASSER server failed, as it was not possible to find any stable secondary or tertiary structures. Taken together, these findings suggest that the linker is indeed rather flexible, most likely unstructured and not very sensitive to sequence variations. It remains to be shown experimentally, if it additionally participates in any protein–protein interactions that could be related to potential Elongator-independent “moonlighting” functions of Kti12.

Our data show that the non-canonical K14L substitution within the Walker A motif decreases nucleotide-binding affinity of Kti12, but does not result in catalytically inactive protein. Kti12 remains active under physiological conditions, as the cytoplasmatic ATP concentration in yeast is close to 2 mM (Koç et al. 2004). We have shown that the *ScKTI12*–*K14L* allele can rescue the tRNA modification phenotype of a Kti12 deficient strain in vivo (Fig. 2b) and that the *Ct*Kti12 and *An*Kti12 proteins have similar in vitro activities as their K14L or L14K variants, respectively (Fig. 3c). Since cytoplasmatic ATP concentration can decrease during starvation, cellular stress or other conditions the low affinity for ATP of Kti12_K14L_would allow to effectively shut down tRNA modification by Elongator via Kti12. Perhaps at a low-energy state of the cell Kti12 deactivation might be required to activate specialized translational programs and the production of Elongator-independent proteomes. If this potential regulatory circuits existed, the question arises whether it represents a unique feature of *Eurotiomycetes* or similar regulatory “bypasses” are present in case of other organisms and other regulatory ATPases.

**Fig. 4:**
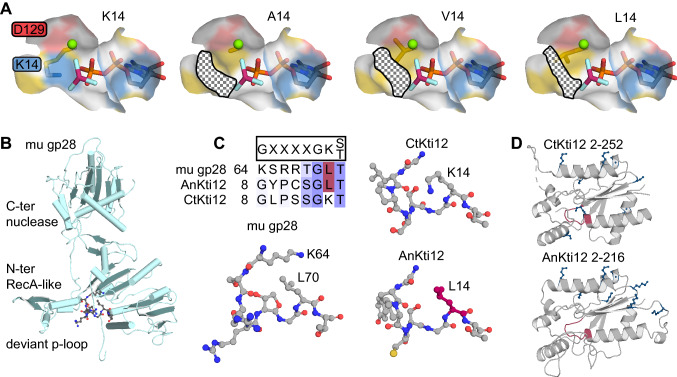
Influence of K14L on the nucleotide binding pocket of Kti12 protein. **a** Hydrophobicity of nucleotide binding pocket of Kti12 mutants. PDB ID: 6QP0. Nucleotide binding pocket consists of positively charged (blue), negatively charged (red) and hydrophobic (yellow) residues. Only surface of the residues within 5 Å from the phosphor atoms of ADP is shown. Kti12 is shown bound to ADP (standard coloring), AlF3 (cyan and pink) and magnesium (green). K14A mutation creates a relatively big gap, leaving the hydrophobic core unprotected. Subsequent K14V and K14L substitutions gradually repair the breach. **b** A homology-based model of gp28 terminase (based on PDB ID 3EZK) from mu phage with previously described deviant Walker-A motif shown in ball and stick representation. **c** Multiple sequence alignment and structural comparison of Walker A motifs in CtKti12, AnKti12 (based on 6QP0) and mu phage gp28. Shades of violet indicate conservation score. Please note that AnKti12 Walker A motif does not have additional lysine which is present in case of mu gp28, but has L14 residue (pink) which corresponds to L70 residue of mu gp28. **d** An overview of lysines (dark blue) present in the ATPase domain of CtKti12 (PDB ID 6QP0, top) and AnKti12 (based on PDB ID 6QP0; bottom). Walker A motif is highlighted in pink

We analyzed the most crucial charged and hydrophobic residues within the *Ct*Kti12 nucleotide-binding pocket and simulated the structural consequences upon mutation of K14. As the side chain of lysine is relatively long, K14A creates a significant breach which may allow water to reach the hydrophobic core of the protein. K14V and K14L substitutions would gradually revert this breach (Fig. 4a).

In addition, we compared available structural information from members of the ‘DxTN’ kinase family, named after its Walker B motif (Leipe et al. 2003). Superposition of ATPase domains of eukaryotic Kti12 (PDB ID: 6QP0), archaeal PSTK from *Methanocaldococcus janashii* (PDB ID: 3ADB), and T4 bacteriophage PNK (PDB ID: 1LY1) revealed highly similar ATPase folds with a particularly strong conservation of the Walker A motif. While the relative position of K14 and the hydrophobic core is almost identical in the analyzed structures, we failed to identify any archaeal or viral proteins carrying L14. Furthermore, we searched the literature for described deviations from Walker A motifs that would include substitution of signature lysine residues by leucine. Gp28, a terminase from phage mu that allows the phage to pack its genome into the capsid, was described to carry a similar substitution (Mitchell and Rao 2004). The authors mentioned that the function of L70 may be successfully complemented by K64 located in close vicinity. We performed homology-based modeling to obtain a model of the gp28 structure (Fig. 4b). We compared Walker A motif of gp28 with those of *Ct*Kti12 and *An*Kti12 in terms of sequence and structure. Kti12 harbors no lysine in a position that would correspond to a previously described K64 residue in gp28 (Fig. 4c). Furthermore, no other lysine from the ATPase domain can perform instead of K14. According to our homology-based model, all lysine residues are located far away from the Walker A motif (Fig. 4d). We would like to highlight that a critical signature substitution within the Walker A motif of *Eurotiomycetes* Kti12 protein may have regulatory importance without diminishing its basal ATPase function. To systematically defy the conservation of the Walker A motif, further bioinformatical research is needed to pinpoint similar substitutions occuring in other kinases and proteomes.

## Electronic supplementary material

Below is the link to the electronic supplementary material.

Supplementary file1 (DOCX 1012 kb)
