# Supplementary material for: Fungal Kti12 proteins display unusual linker regions and unique ATPase p-loops
Source: Curr Genet. 2020 Mar 31;66(4):823–33. doi: 10.1007/s00294-020-01070-2 (PMC7363723; doi:10.1007/s00294-020-01070-2)
Supplement: Supplementary file 1 — Supplementary file1 (DOCX 1012 kb) [file 294_2020_1070_MOESM1_ESM.docx]

Supplementary Information

Fungal Kti12 proteins display unusual linker regions and unique ATPase p-loops

Rościsław Krutyhołowa^1,2,*^, Annekathrin Reinhardt-Tews^3,*^, Andrzej Chramiec-Głąbik^1^, Karin D Breunig^3,#^ and Sebastian Glatt^1,#^

^1^ Malopolska Centre of Biotechnology (MCB), Jagiellonian University, Krakow, Poland

^2^ Faculty of Biochemistry, Biophysics and Biotechnology, Jagiellonian University, Krakow, Poland.

^3^ Institut für Biologie, Martin-Luther-Universität Halle-Wittenberg, Halle (Saale), Germany

* these authors contributed equally

# Correspondence to KDB ([karin.breunig@serymun.com](mailto:karin.breunig@serymun.com)) and SG ([sebastian.glatt@uj.edu.pl](mailto:sebastian.glatt@uj.edu.pl))

ORCIDs: RK - 0000-0002-8200-5627, ART - 0000-0002-0295-0312, ACG - 0000-0002-3544-7211, KDB - 0000-0002-0319-3114, SG - 0000-0003-2815-7133.

Key words: Kti12, PSTK, flexible linker, active site, P-loop, ATPase

Running title: Detailed analyses of the active site and linker regions in Kti12

**
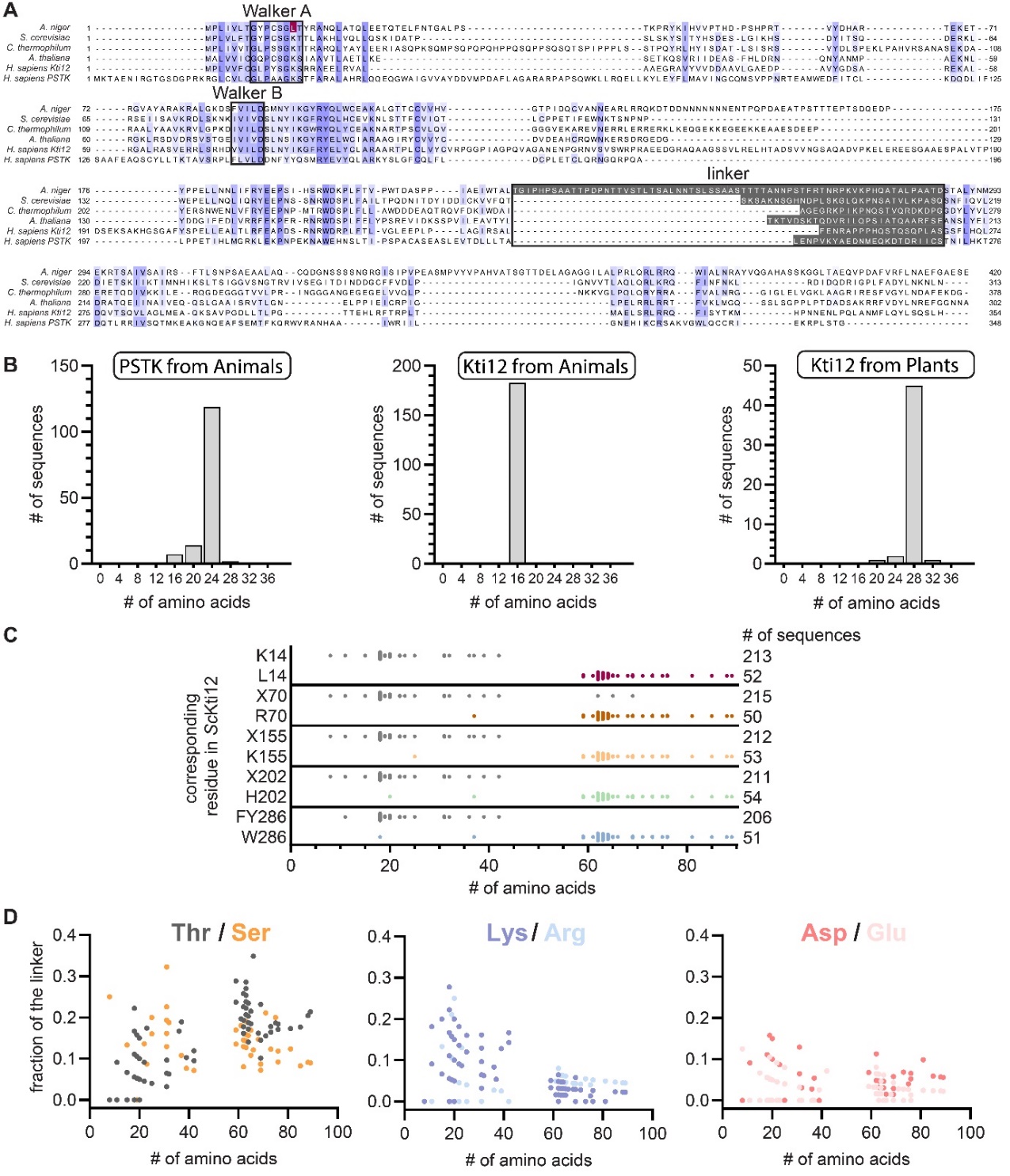
**

**Figure S1. Analysis of Kti12 linker properties.**

(A) Representative multiple sequence alignment of Kti12 proteins from *Aspergillus niger*, *Saccharomyces cerevisiae*, *Chaetomium thermophilum*, *Arabidopsis thaliana*, *Homo sapiens* and *Homo sapiens* PSTK. Shades of blue indicate conservation score evaluated using BLOSUM62 matrix. Walker A, Walker B and the linker are highlighted with black rectangles. L14 in *A. niger* Kti12 is highlighted with pink. (B) PSTK and Kti12 linker length distribution across non-fungal eukaryotic kingdoms of life. Numbers on O_x_ indicate center of the basket, for example, 16±2. (C) Classification results for other high-scored residues that enable separation of sequences with short (grey) and long (colored) linker. X indicates any amino acid. Number of sequences harboring particular residue is shown on the right. (D) Content analyses of the relative presence of particular amino acids in fungal Kti12 linkers.

**
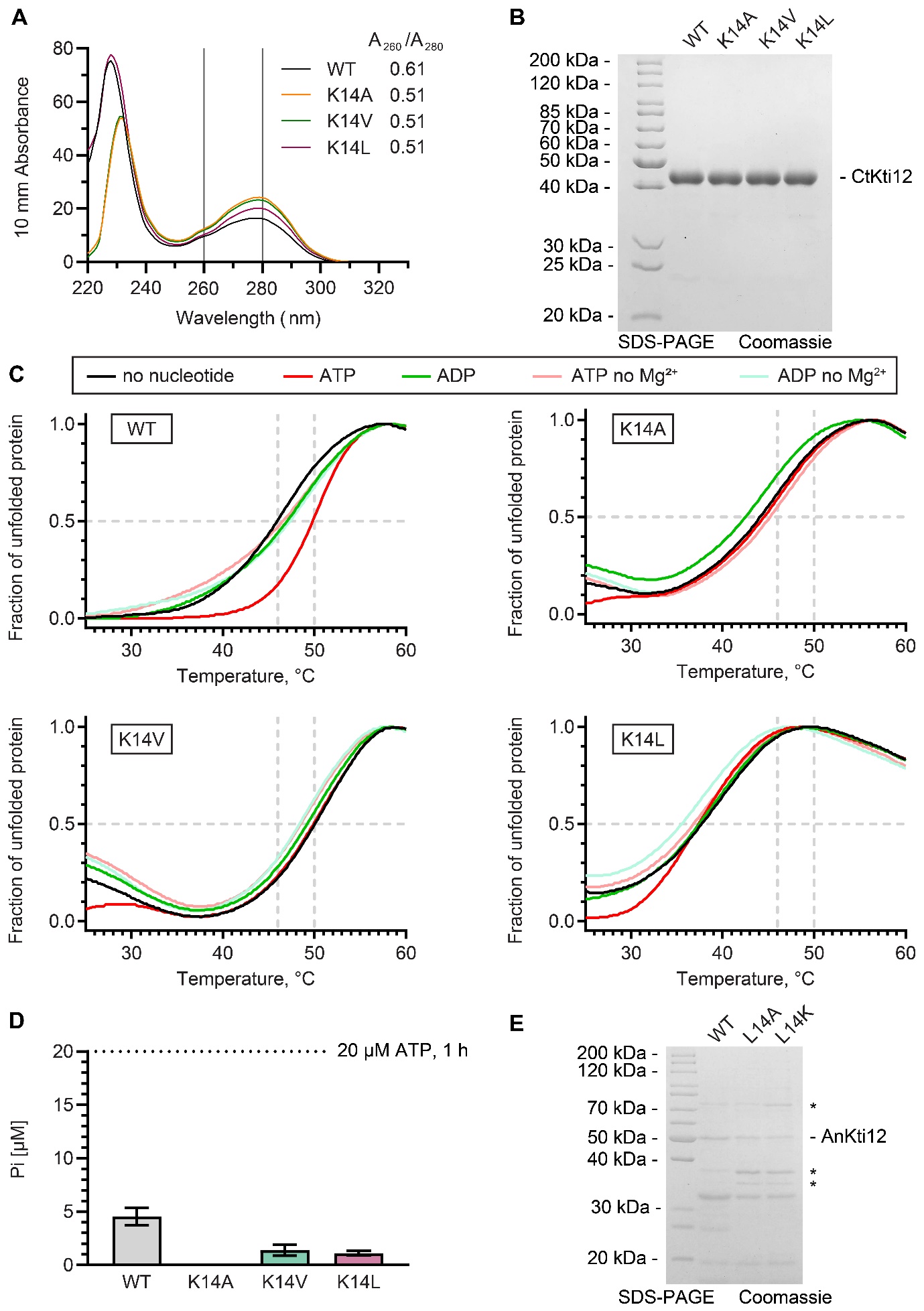
**

**Figure S2. Analysis of protein purity, extended thermal shift data and ATP-hydrolysis at semi saturating conditions.**

(A) UV absorption spectra of Kti12 proteins. A260/A280 ratios indicate, that all mutant samples do not contain any nucleotide contamination. (B) SDS-PAGE separation of *Ct*Kti12 mutants. Purified proteins are free from any protein contaminations. (C) Detailed overview of thermal shift profiles of Kti12 mutants stimulated with nucleotides. Sample without addition of nucleotide (black) is used as a reference to evaluate effect of 1 mM ADP (green) or 1 mM ATP (red). Pale curves indicate samples deprived of magnesium. Vertical lines illustrate WT thermos-stability without addition of nucleotide (left) and upon stimulation with ATP (right). Curves represent an average of three independent experiments. (D) To validate nucleotide affinity data enzymatic activity of Kti12 mutants was evaluated in semi-saturating substrate concentration (20 μM ATP). ATPase activity was elicited using tRNA^Sec^, level of free phosphate was measured using malachite green assay after 1 h incubation at 37 °C. (E) 12% SDS-PAGE separation of *Aspergillus niger* Kti12 mutants. Asterisks highlight contaminations that are present in mutant proteins and not present in the WT AnKti12.

**Table S1. *Saccharomyces cerevisiae* strains.**

| Strain | Relevant genotype | Description | Source |
| --- | --- | --- | --- |
| UMY2893 | *MATα SUP4 trp1-1*  *leu2-3,112*  *can1-100 ura3-1*  *ade2-1 his3-11,15* | Tester strain for nonsense suppression by SUP4 encoded suppressor tRNA^Tyr^ *ade2*-*1* and *can1*-*100* alleles contain ochre nonsense codons. | Huang et al., 2005 |
| KDY2 | *Scelp3Δ::KlURA3* | Disruption of *ELP3* gene in UMY2893 background | Dieckow, Katja,  Breunig Lab, Halle 2011 |
| RZY144 | *Sckti12-K14A-HA_6_ ::KlTRP1* | UMY2893 with tagged variants of *ELP1* and *kti12-K14A*::*TRP1* | Krutyhołowa et al., 2019 |
| CKY40 | UMY2893, *Sckti12Δ::KlURA3* | Disruption of *KTI12* gene in UMY2893 background, parent strain for *kti12* mutants | This study |
| ART5 | CKY40*,*  *Sckti12*-*K14L* | Isogenic with CKY40 but replacement of *kti12::URA3* by *kti12-K14L* allele | This study |
| ART6 | CKY40*,*  *AnKTI12* | Isogenic with CKY40 but replacement of *kti12::URA3 by AnKTI12* gene from *Aspergillus niger* | This study |
| ART7 | CKY40*, Sckti12Δknot* | Isogenic with CKY40 but replacement of *kti12::KlURA3 by ScKTI12-Δk* allele encoding Kti12 variant with deleted knot (aa 184 – 196Δ) | This study |
| ART8 | CKY40*, Sckti12Δlinker* | Isogenic with CKY40 but replacement of *kti12::KlURA3* by *ScKTI12-Δl* allele encoding Kti12 variant with aa 197 – 213 (linker part) deleted | This study |
| ART9 | CKY40*, Sckti12Δ184-213* | Isogenic with CKY40 but replacement of *kti12::KlURA3 by ScKTI12-Δkl* allele encoding Kti12 variant with knot and linker (aa 184 – 213) deleted | This study |
| ART10 | CKY40*,*  *Sckti12-NTD* | Isogenic with CKY40 but replacement of *kti12::KlURA3 by ScKTI12-NTD* expressing only the Kti12 N-terminal domain (aa 1 - 184) | This study |
| ART11 | CKY40*,*  *Sckti12-AnKnoLi* | Isogenic with CKY40 but replacement of *kti12::KlURA3* by *ScKTI12-AnKNOLI* allele resulting in exchange of ScKti12 linker (aa 183-212) with corresponding region from *A. niger KTI12* | This study |
| ART15 | CKY40*,*  *Sckti12*-*K14V* | Isogenic with CKY40 but replacement of *kti12::KlURA3* by *kti12-K14V* allele | This study |
